# The effect of SNPs in lncRNA as ceRNA on the risk and prognosis of hepatocellular carcinoma

**DOI:** 10.1186/s12864-022-09010-9

**Published:** 2022-11-24

**Authors:** Han Mo, Xi Wang, Guohua Ji, Xiao Liang, Yi Yang, Wenjing Sun, Xueyuan Jia, Lidan Xu, Yuandong Qiao, Henan Zhou, Wenhui Zhao, Songbin Fu, Xuelong Zhang

**Affiliations:** 1 Laboratory of Medical Genetics, Harbin Medical University, Harbin, 150081 China; 2 Key Laboratory of Preservation of Human Genetic Resources and Disease Control in China (Harbin Medical University), Ministry of Education, Harbin, 150081 China; 3 Department of Gastroenterology, the First Affiliated Hospital, Harbin Medical University, Harbin, 150001 China; 4 Department of Clinical Laboratory, Harbin Medical University Cancer Hospital, Harbin, 150081 China; 5 Department of Internal Medicine, Harbin Medical University Cancer Hospital, Harbin, 150081 China

**Keywords:** HOTAIR, PVT1, EGFR-AS1, HCC, Risk, Prognosis, SNP, ceRNA

## Abstract

**Background:**

Most susceptible loci of hepatocellular carcinoma (HCC) identified by genome-wide association studies (GWAS) are located in non-coding regions, and the mechanism of action remains unclear. The objective of this study was to explore the association of single nucleotide polymorphisms (SNPs) on long non-coding RNAs (lncRNAs) that affect competing endogenous RNAs (ceRNA) regulation mechanism with the risk and prognosis of HCC.

**Methods:**

Based on a set of bioinformatics strategies, eight lncRNA genes that affect HCC through the mechanism of lncRNA-mediated ceRNA were systematically screened, and 15 SNPs that affect microRNA (miRNA) binding in these lncRNA genes were annotated. Genotyping was performed in 800 HCC cases and 801 healthy controls to examine associations of these SNPs with HCC in a northeastern Chinese Han population.

**Results:**

The GG, GC and GG + GC genotypes of *HOTAIR* rs7958904 were associated with a 0.65, 0.59 and 0.63-fold decreased HCC risk, respectively. In addition, HCC patients with *PVT1* rs3931282 AA + GA genotypes were less prone to develop late-stage cancers in a stratified analysis of clinical characteristics. When stratified by clinical biochemical indexes, rs1134492 and rs10589312 in *PVT1* and rs84557 in *EGFR-AS1* showed significant associations with aspartate aminotransferase (AST), alanine aminotransferase (ALT) or AST/ALT ratio in HCC patients. Furthermore, we constructed potential ceRNA regulatory axes that might be affected by five positive SNPs to explain the causes of these genetic associations.

**Conclusions:**

*HOTAIR* rs7958904, *PVT1* rs3931282, rs1134492 and rs10589312, and *EGFR-AS1* rs84557 might be predictors for HCC risk or prognosis. Our results provide new insights into how SNPs on lncRNA-mediated ceRNAs confer interindividual differences to occurrence and progression of HCC.

**Supplementary Information:**

The online version contains supplementary material available at 10.1186/s12864-022-09010-9.

## Background

According to the Global Cancer Statistics 2020, primary liver cancer is the sixth most common cancer and the third leading cause of cancer mortality worldwide (new cases, 906,000/y; deaths, 830,000/y) [1]. The most common histology (about 80%) of primary liver cancer is hepatocellular carcinoma (HCC) [2]. Notably, East Asia is the region with the highest risk of HCC, with more than 50% of the world's HCC cases coming from China [3]. Hepatocarcinogenesis is a multi-step process involving multiple risk factors in its occurrence, promotion and development [4]. Genetic variation plays an important role in these risk factors. In the past few decades, more and more genome-wide association studies (GWAS) have been applied to identify HCC susceptibility single nucleotide polymorphisms (SNPs) [5], most of which are located in non-coding regions [6]. Mutations in regulatory regions may lead to subtle changes in gene expression in cell type or tissue-specific manner, which may predispose mutation carriers to changes in cancer susceptibility throughout their life cycle [7–9]. Therefore, the mechanism of non-coding SNPs on HCC can be explained from the perspective of expression regulatory.

Long non-coding RNAs (lncRNAs) are defined as a class of transcripts with more than 200 nucleotides, which participate in transcription, post transcription, post-translational regulation and intercellular signal transduction [10]. LncRNAs serve as critical regulators of regulators of tumorigenesis and metastasis [11, 12]. MicroRNAs (miRNAs) are another small non-coding RNA molecules with a nucleotide length of about 22 that mainly mediate the process of post-transcriptional gene silencing [13]. As competing endogenous RNAs (ceRNAs), lncRNAs interact with miRNAs through complementary sequences and become the bait or sponge of miRNAs, which has been widely confirmed by functional studies [14–16]. With the development of high-throughput technologies, an increasing number of cancer-related studies have been published using The Cancer Genome Atlas (TCGA). HCC related lncRNA-mediated ceRNA networks have also been reported in recent TCGA-based articles [17].

It is well known that SNPs located in lncRNA-miRNA binding regions may affect their interactions, thereby altering their functions and leading to genome-wide butterfly effects [18]. Several evidences have indicated that these SNPs are involved in the occurrence and development of various cancers. For example, one study showed that rs2147578 CG and GG genotypes were significantly associated with an increased risk of colorectal cancer by influencing the combination of lnc-LAMC2-1:1 and miR-128-3p [19]. Another study confirmed that the LINC00673 polymorphism created a miR-1231 binding site and affected the risk of pancreatic cancer by interfering with PTPN11 degradation [9]. Encouragingly, a large number of SNPs located in lncRNA-miRNA binding regions have recently been identified by the lncRNASNP2 database, which is also supported by experimental evidence [20]. These SNPs are more likely to play a role by interfering with the ceRNA function of lncRNAs.

Thus, we suggest that SNPs affecting lncRNA-miRNA interaction may cause HCC risk. To further test the hypothesis, we systematically screened out such potentially functional SNPs with a set of bioinformatics strategies. Next, a case–control study was conducted in a northeastern Chinese Han population to assess the relationship of these candidate SNPs with the occurrence and development of HCC. Finally, we discussed how positive SNPs affect HCC through the potential regulatory axis of ceRNA.

## Results

### Differential expression analysis

**Fig. 1:**
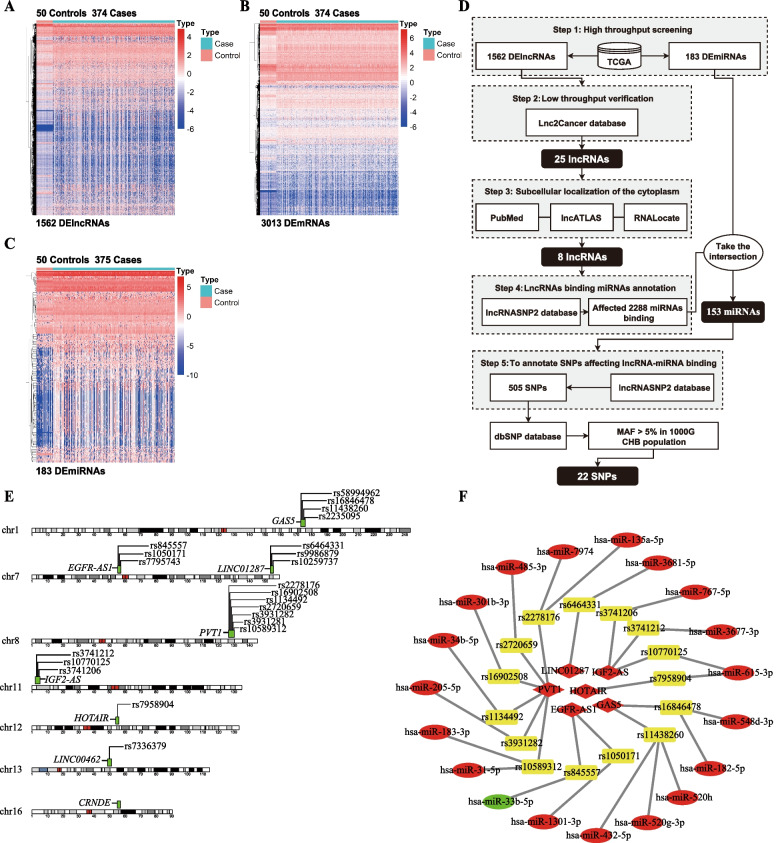
Screening results of lncRNA related SNPs. **A** Clustering heatmap of DElncRNAs. **B** Clustering heatmap of DEmRNAs. **C** Clustering heatmap of DEmiRNAs. **D** Flow chart of screening lncRNA related SNPs. In the heat map, red represents high expression and blue represents low expression; pink represents the paracancerous control group and blue represents the HCC case group. **E **Genomic visualization of 8 lncRNAs and 22 candidate SNPs. **F** Related lncRNA-SNP-miRNA network diagram. Yellow rectangles are SNPs, red is high expression, green is low expression, ellipses are miRNAs, and diamonds are lncRNAs

The gene expression heatmaps of differentially expressed lncRNAs (DElncRNAs), differentially expressed mRNAs (DEmRNAs) and differentially expressed miRNAs (DEmiRNAs) were shown in Fig. 1A, Fig. 1B and Fig. 1C, respectively. Based on differential analysis, we obtained 1562 DElncRNAs (1415 up-regulated and 147 down-regulated), 3013 DEmRNAs (2533 up-regulated and 480 down-regulated) and 183 DEmiRNAs (175 up-regulated and 8 down-regulated).

### The screening of 22 candidate SNPs on HCC related ceRNAs

As shown in the flow chart (Fig. 1D), 22 candidate SNPs on eight HCC related lncRNA mediated ceRNAs were screened out following steps below. Step1 and step 2: 1562 DElncRNAs screened by high throughput were validated by low throughput experiments in HCC samples using Lnc2Cancer database (http://bio-bigdata.hrbmu.edu.cn/lnc2cancer/), 25 of which had consistent expression trends and passed the validation. Step 3: Considering that the ceRNA mechanism of lncRNAs mainly occurred in the cytoplasm, based on PubMed database and web tools RNALocate (https://www.rna-society.org/rnalocate/) and lncATLAS (https://lncatlas.crg.eu/), eight of 25 lncRNAs were retrieved with evidence of subcellular localization in the cytoplasm. Step 4 and step 5: The lncRNASNP2 database was used to annotate eight lncRNAs, and 2699 SNPs affecting the binding of lncRNA-miRNA were extracted. These 2699 SNPs affected the binding of 2288 miRNAs, but only 153 miRNAs were retained through DEmiRNAs filtering, involving 505 SNPs in total. Finally, based on the dbSNP database and the standard of MAF > 0.05 in the Han Chinese in Beijing (CHB) population of the 1000 Genomes Project, 22 of 505 SNPs were screened out. Genomic visualization of eight lncRNAs and 22 candidate SNPs were shown in Fig. 1E.

### Identification of 15 SNPs for genotyping

As described in the Method section, the feasibility of genotyping for 22 candidate SNPs was evaluated. In the 48-Plex SNPscan™ typing system, 22 SNPs were classified into four levels: first, second, third and fail (Additional file 1: Supplementary Fig. 1A). Subsequent genotyping was abandoned for unqualified grade SNPs (rs7336379, rs9986879 and rs10259737), which were unable to design primers for genotyping. Haploview 4.2 software (https://sourceforge.net/projects/haploview/) was used to determine the linkage disequilibrium (LD) by the standardized D’ and r^2^ values for the other 19 SNPs. Reference population is CHB of the 1000 Genomes Project. The results showed strong LD (r^2^ > 0.8) between some SNPs (Additional file 1: Supplementary Fig. 1B). Specifically, rs1050171 on *EGFR-AS1* can represent rs7795743, rs16846478 on *GAS5* can represent rs58994962 and rs2235095, and rs3931282 on *PVT1* can represent rs3931281 for subsequent genotyping. Finally, a total of 15 SNPs were genotyped, and their network diagram with corresponding lncRNAs and miRNAs was shown in Fig. 1F.

### Characteristics of the study population

The demographic characteristics of 1601 subjects, clinical information and clinical test indexes of 800 HCC patients enrolled in our study are shown in Table 1. No significant differences were observed for age, gender and cigarette smoking between cases and controls (all *P* > 0.05). The P-P plot (Additional file 1: Supplementary Fig. 2) and Levene’s test (Additional file 2: Supplementary Table 1) respectively show that the age conforms to the normal distribution and has homogeneity of variance. However, alcohol drinking was significantly different between the two groups (*P* = 0.010). Whether the difference was significant or not, these demographic characteristics were included in the statistical model as potential confounding factors for genetic association analysis.

**Table 1:** Characteristics of the study population

Variable	Cases (*N* = 800)	Controls (*N* = 801)	*P* value
Age (years)	Mean ± S.D	Mean ± S.D	
	56.87 ± 10.25	56.97 ± 10.26	0.851^a^
Gender			
Male	652 (81.5%)	688 (86.0%)	
Female	148 (18.5%)	133 (16.6%)	0.319^b^
Cigarette smoking			
No	415 (51.9%)	430 (53.8%)	0.469^b^
Yes	385 (48.1%)	371 (46.4%)	
Alcohol consumption			
No	463 (57.9%)	514 (64.3%)	***0.010***^b^
Yes	337 (42.1%)	287 (35.9%)	
Family history			
No	757 (94.6%)	-	-
Yes	43 (5.4%)	-	-
HBsAg			
Negative	171 (21.4%)	-	-
Yes	629 (78.6%)	-	-
Anti-HCV			
Negative	697 (87.1%)	-	-
Yes	103 (12.9%)	-	-
Liver cirrhosis			
No	230 (28.8%)	-	-
Yes	570 (71.3%)	-	-
Child–Pugh grade			
A	696 (87.1%)	-	-
B or C	104 (12.9%)	-	-
Tumor size			
< 5 cm	418 (52.3%)	-	-
≥ 5 cm	382 (47.8%)	-	-
Tumor number			
Solitary	400 (50.0%)	-	-
Multiple	400 (50.0%)	-	-
Vascular invasion			
No	541 (67.6%)	-	-
Yes	259 (32.4%)	-	-
Lymphatic metastasis			
No	614 (76.8%)	-	-
Yes	186 (23.3%)	-	-
Distant metastasis			
No	631 (78.9%)	-	-
Yes	169 (21.1%)	-	-
TNM staging			
I + II	275 (34.4%)	-	-
III + IV	525 (65.6%)	-	-

The bold and italicized value indicates statistical significance

^a^ Student’s *t*-test

^b^ Chi-square test. *S.D.* standard deviation

## The association of 14 candidate SNPs in lncRNA-mediated ceRNAs with HCC risk

**Fig. 2:**
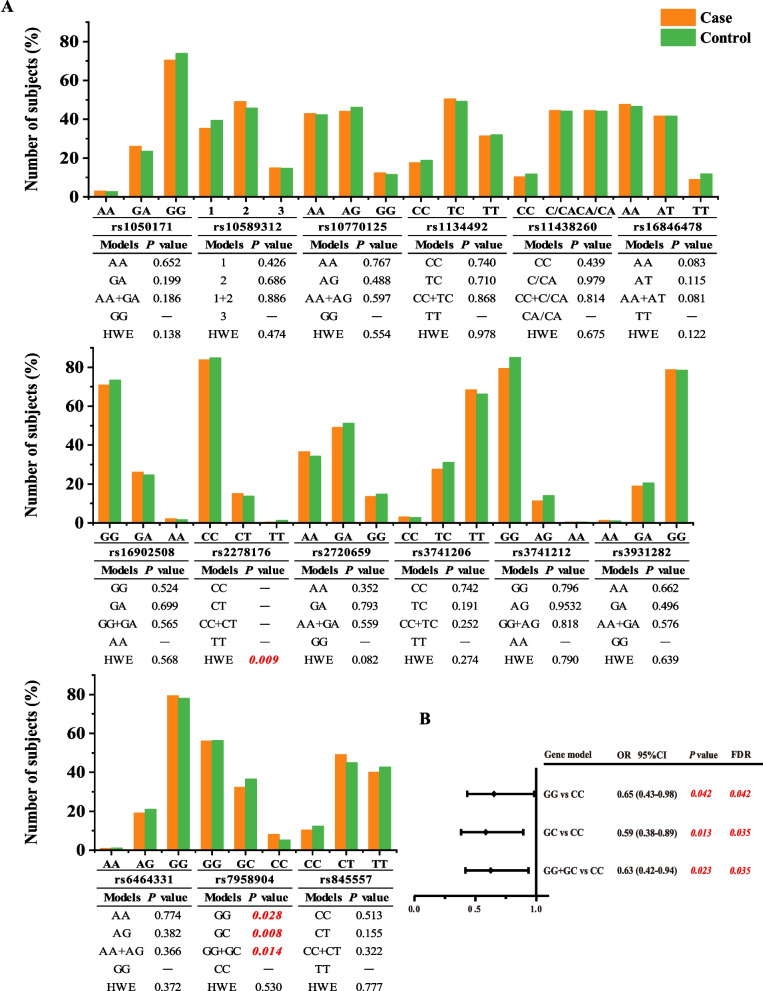
The genotype distribution and association analysis of 15 lncRNA-SNPs with HCC risk. **A** The genotype distribution, HWE test, and association analysis under the codominant model 1, codominant model 2 and dominant model. 1 is the TCTTGC/TCTTGC genotype of rs10589312, 2 is the TCTTGC/T genotype of rs10589312, 1 + 2 is the TCTTGC/TCTTGC + TCTTGC/T genotype of rs10589312, and 3 is the T/T genotype of rs10589312. The values in red italics are statistically significant. **B** Association analysis of SNP rs7958904 with HCC risk after adjusted. OR and *P* values were adjusted for age, gender, smoking and drinking by logistic regression. The values in red italics are statistically significant

The genotype distribution of 15 SNPs is shown in Fig. 2A. Rs2278176 was excluded in subsequent analysis because it did not conform to Hardy–Weinberg equilibrium (HWE). Further analysis was conducted on the effect of the genotypes of 14 SNPs under different genetic models (Fig. 2 and Additional file 3: Supplementary Table 2). As shown in Fig. 2A, compared with the CC genotype, the distribution of GG, GC and GG + GC genotypes of rs7958904 was significantly different between the case and control group (GG vs CC, *P* = 0.028, codominant model 1; GC vs CC, *P* = 0.008, codominant model 2; GG + GC vs CC, *P* = 0.014, dominant model). Then binary logistic regression was used to explore the association between rs7958904 and HCC risk. Age, gender, cigarette smoking and alcohol drinking were included as covariates to adjust for confounders. As shown in Fig. 2B, compared with CC genotype, a protective effect of rs7958904 GG, GC and GG + GC genotypes was found for HCC risk (*P* = 0.042, OR = 0.65, 95% CI 0.43–0.98; *P* = 0.013, OR = 0.59, 95% CI 0.38–0.89; and *P* = 0.023, OR = 0.63, 95% CI 0.42–0.94, respectively). Furthermore, the association still stood after an FDR correction (corrected *P* = 0.042; *P* = 0.035 and *P* = 0.035, respectively). For other SNPs, no association with HCC risk was observed in any genetic model (Fig. 2 and Additional file 3: Supplementary Table 2).

## Association analysis between 14 SNPs and clinical characteristics of HCC patients

**Fig. 3:**
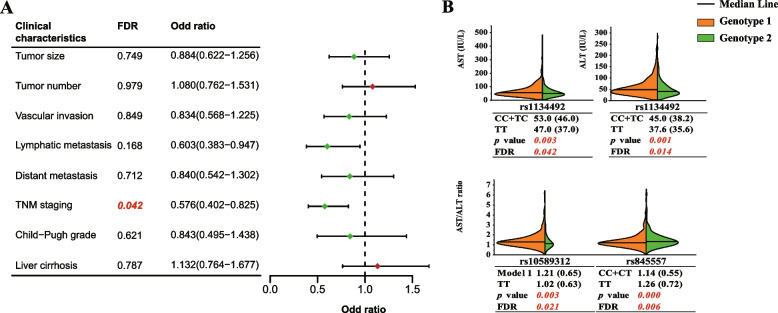
Association analysis of lncRNA-SNPs with clinical characteristics or clinical test indexes in HCC patients. **A** Association analysis of rs3931282 with clinical characteristics in HCC patients. **B **Significant results of association analysis between 14 SNPs and clinical test indicators. Model 1 is the TCTTGC/TCTTGC + TCTTGC/T genetic model of rs10589312. Model 2 is the CC + C/CA genetic model of rs11438260. OR and *P* values were adjusted for age, gender, smoking, drinking, HBsAg and anti-HCV by logistic regression. The values in red italics are statistically significant. The size of the clinical test indexes is represented by the median value (inter quartile range)

The association between 14 SNPs and clinical characteristics of HCC patients, including liver cirrhosis, Child–Pugh grade, tumor size, tumor number, vascular invasion, lymphatic metastasis, distant metastasis and TNM stage, was analyzed by binary logistic regression. And age, gender, cigarette smoking, alcohol drinking, family history, HBsAg and anti-HCV were used as covariates to adjust for confounding factors. FDR correction was performed for all *P* values. The HCC patients were staged according to the AJCC-TNM classification [21]. Early-stage patients included patients with stage I and II. Advanced stage patients included patients with stage III and IV. As shown in Fig. 3A, the distribution of rs3931282 genotypes showed a significant difference between early stage and advanced stage. In detail, compared with GG genotype, the frequency of AA + GA genotype was higher in early stage than that in advanced stage and showed a decreased risk for HCC progression (FDR = 0.042, OR = 0.576, 95% CI 0.402–0.825). In addition, the results of associations of other SNPs with clinical characteristics are shown in Additional file 4: Supplementary Table 3.

## Association analysis between 14 SNPs and clinical test indexes of HCC patients

In this study, the levels of α-fetoprotein (AFP), AST, ALT and AST/ALT ratio in HCC patients did not conform to the normal distribution. Mann–Whitney U test was used to explore the correlation between the distribution of 14 SNP genotypes and these indicators. FDR correction was performed for all *P* values at the same time. As shown in Fig. 3B, the AST and ALT levels in patients with rs1134492 CC + TC genotype were significantly higher than those with TT genotype (corrected *P* = 0.042 and 0.014, respectively). Meanwhile, AST/ALT ratios were significantly higher in patients with rs10589312 TCTTGC/TCTTGC + TCTTGC/T genotype than those with TT genotype (corrected *P* = 0.021), and AST/ALT ratios were significantly lower in patients with rs84557 CC + CT genotype than those with TT genotype (corrected *P* = 0.006). In addition, the results of associations of other SNPs with clinical test indexes are shown in Additional file 1:  Supplementary Fig. 3.

### Acquisition of mRNAs targeted by miRNAs

**Fig. 4:**
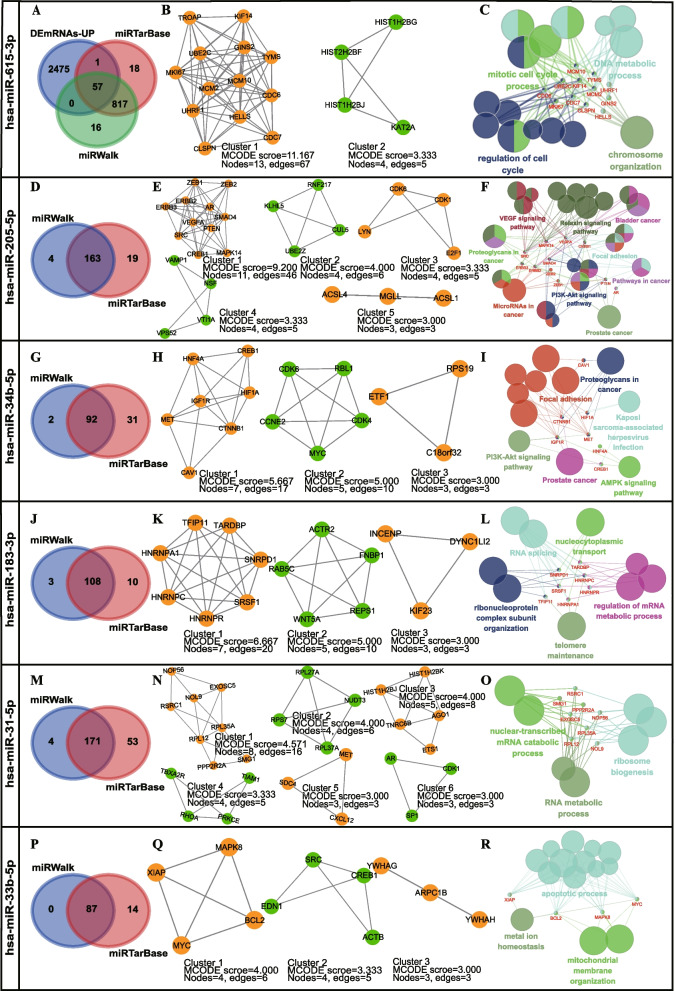
Bioinformatics analysis of positive SNPs-related miRNAs. **A**-**C** Bioinformatics analysis of *HOTAIR*-rs7958904-miR-615-3p. **D**-**F** Bioinformatics analysis of *PVT1*-rs3931282-miR-205-5p. **G**-**I** Bioinformatics analysis of *PVT1*-rs1134492-miR-34b-5p. **J**-**L** Bioinformatics analysis of *PVT1*-rs10589312-miR-183-3p. **M**–**O** Bioinformatics analysis of *PVT1*-rs10589312-miR-31-5p. **P**-**R** Bioinformatics analysis of *EGFR-AS1*-rs84557-miR-33b-5p. Each node in enrichment analysis of functional modular genes represents a term, the connection between the node and gene reflects the existence of correlation, and the color reflects the enrichment classification of node and gene

In this association study, five SNPs (rs7958904, rs3931282, rs1134492, rs10589312 and rs84557) were found to be associated with the occurrence and prognosis of HCC. These five loci located on lncRNA coding genes would affect the binding of six microRNAs (miR-615-3p, miR-205-5p, miR-34b-5p, miR-183-3p, miR-31-5p and miR-33b-5p) to lncRNAs (Fig. 1E). The target genes of these six miRNAs were obtained through screening mRNAs shared by miRWalk and miRTarBase databases. For miR-615-3p, highly expressed DEmRNAs in cancers were also compared with candidate target mRNAs to obtain cross-genes between them. Finally, 57 target genes of miR-615-3p, 163 target genes of miR-205-5p, 92 target genes of miR-34b-5p, 108 target genes of miR-183-3p, 171 target genes of miR-31-5p and 87 target genes of miR-33b-5p were extracted (Fig. 4A, Fig. 4D, Fig. 4G, Fig. 4J, Fig. 4M and Fig. 4P, respectively).

### The modular analysis of PPI network of miRNA-target mRNAs

Firstly, PPI networks of six miRNAs targets were constructed based on String, and the Cytoscape plug-in MCODE was used for module analysis. Then, the PPI networks of miR-615-3p, miR-205-5p, miR-34b-5p, miR-183-3p, miR-31-5p and miR-33b-5p target genes were constructed into two, five, three, three, six and three functional modules, respectively. (Fig. 4B, Fig. 4E, Fig. 4H, Fig. 4K, Fig. 4N and Fig. 4Q, respectively). Furthermore, KEGG or Go-BP enrichment of the most important modules was performed using Cytoscape plug-ins ClueGO and CluePedia, which was the first cluster in the PPI network module analysis of six miRNAs target genes. The results of KEGG or Go-BP enrichment analysis were shown in Fig. 4C, Fig. 4F, Fig. 4I, Fig. 4L, Fig. 4O and Fig. 4R. Therefore, the targets of the above miRNAs may be involved in the occurrence and development of HCC by participating in pathways or biological processes shown in the figure.

## Discussion

In this study, eight lncRNA-mediated ceRNAs associated with HCC were screened by bioinformatics methods. Compared with recent researches on screening HCC related ceRNA networks based on TCGA, such as the study of Ye et al. [22], the screening process of this study has significant characteristics on the basis of the same scientific nature. We not only used the Lnc2Cancer database to extract the lncRNAs supported by the low throughput experimental verification, but also considered the subcellular localization of lncRNAs. The latter is due to the fact that the biological process of lncRNA competing with mRNA to bind miRNA mainly occurs in the cytoplasm [23]. Then eight lncRNAs were annotated and the functional SNPs that affect the binding of miRNAs to lncRNAs were extracted. After evaluating these SNPs, 15 SNPs were finally selected and a case–control study was conducted in a Han Chinese population to explore the potential associations between these candidate SNPs and the risk of HCC.

In the association analysis, five positive SNPs that were significantly associated with susceptibility or prognosis of HCC were identified. To our knowledge, this study first reported the relationship between rs3931282, rs1134492, rs10589312 and rs84557 and the prognosis of HCC. In HCC cohort, the TNM stage of AA + GA genotype of *PVT1* rs3931282 was earlier than that of GG genotype, suggesting that the former had a better prognosis. In addition, our results showed that *PVT1* rs1134492 genotypes were associated with AST and ALT levels, while *PVT1* rs10589312 and *EGFR-AS1* rs84557 were associated with AST/ALT ratios of HCC patients. Clinical biochemical indicators often reflect the liver function of patients. For example, the higher the AST and ALT level and AST/ALT ratio, the more serious the hepatocyte injury, the worse the liver function and the worse the prognosis. These results indicated that some promising SNPs of ceRNAs could be used as important cancer biomarkers and contribute to the individualized prognosis of HCC in a specific population.

This study is the first to show that *HOTAIR* rs7958904 was associated with HCC susceptibility, although it has been reported to be associated with the risk of other cancers. In a case–control study of South Korean population [24], individuals with rs7958904 GG genotype were found to have a lower risk of developing colorectal cancer compared to those with CC + GC and GC genotypes. This trend of increased cancer risk of allele C is consistent with that observed in our study. The present study showed that SNP rs7958904 was strongly associated with the HCC risk. Compared with CC genotypes, individuals with rs7958904 GG or GC genotypes showed 0.65-fold or 0.59-fold decreased HCC risk. Therefore, rs7958904 may serve as a promising predictor for HCC risk. However, in a study linking rs7958904 with ovarian cancer in a southern Chinese population, the CC genotype showed a protective effect [25]. One of the reasons for this contradiction may be that the regulatory axis of ceRNA affected by rs7958904 is different in different types of cancers. Therefore, in order to further explain how the five positive SNPs are related to the risk and prognosis of HCC through ceRNA mechanism, the potential regulatory axis of HCC related ceRNA affected by positive SNPs was constructed through miRNA target gene prediction and functional module analysis.

**Fig. 5:**
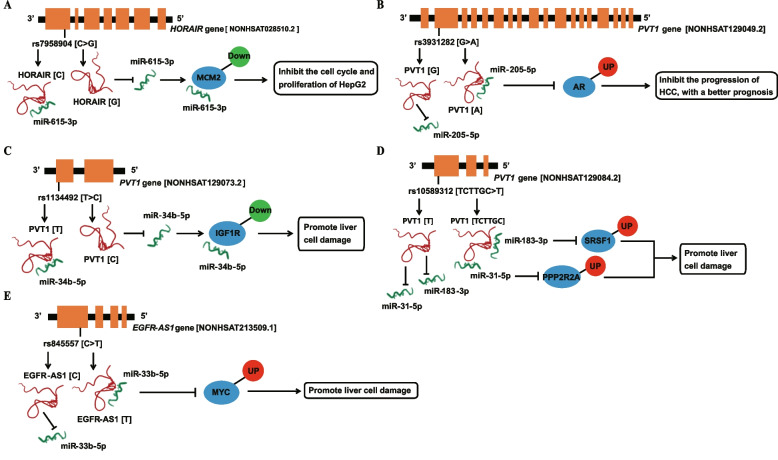
Six potential ceRNA regulatory axes affected by five positive SNPs

The first regulation axis of HCC related ceRNA was affected by the positive SNP rs7958904. As shown in Fig. 5A, according to the annotation of lncRNASNP2 database, the rs7958904 G allele of *HOTAIR* will disrupt the adsorption of *HOTAIR* to miR-615-3p, thereby increasing the silencing effect of miR-615-3p on its target genes. Among the targets of miR-615-3p, inhibition of MCM2 expression could significantly inhibit HepG2 proliferation and cell cycle through the cyclin D-dependent kinases (CDKs) 2/7 pathway [26]. The regulatory axis expounds the possible reason why individuals with *HOTAIR* rs7958904 GG and GC genotypes show a lower risk of developing HCC than those with CC genotype. The second regulation axis of HCC related ceRNA was affected by the positive SNP rs3931282. As shown in Fig. 5B, the rs3931282 A allele of *PVT1* will enhance the adsorption of *PVT1* to miR-205-5p, thereby reducing the silencing effect of miR-205-5p on its target. Among the targets of miR-205-5p, the increased expression of AR gene can increase HCC cell adhesion and inhibit HCC cell migration by activating AR-β1-integrin-AKT signal transduction [27, 28]. This is the possible reason why HCC patients with rs3931282 AA + GA genotype have earlier TNM staging and better prognosis than those with GG genotype.

The third, fourth and fifth regulation axes of HCC related ceRNA were affected by the positive SNP rs1134492, rs10589312 and rs84557, respectively. Specifically, in the third regulatory axis, the rs1134492 C allele of *PVT1* will destroy the adsorption of *PVT1* to miR-34b-5p, thus increasing the silencing effect of miR-34b-5p on its target. Among the targets of miR-34b-5p we excavated, the IGF1R inhibition can promote Fas-induced liver injury [29] (Fig. 5C). In the fourth regulatory axis, the rs10589312 TCTTGC allele of *PVT1* will enhance the adsorption of *PVT1* to miR-183-3p and miR-31-5p, and thus weaken the inhibition of miR-183-3p and miR-31-5p on their targets SRSF1 and PPP2R2A. Among them, SRSF1 overexpression can promote liver injury induced by caspase-dependent apoptosis pathway [30, 31]. Overexpression of PPP2R2A can inhibit Akt phosphorylation, thus blocking the PI3K/Akt signaling pathway and causing liver injury [32, 33] (Fig. 5D). In the fifth regulatory axis, the rs84557 T allele of *EGFR-AS1* will enhance the adsorption of *EGFR-AS1* to miR-33b-5p, thereby reducing the inhibition of miR-33b-5p on its target MYC. MYC overexpression can activate the p14ARF/MDM2 pathway, thereby stimulating p53-mediated apoptosis and promoting liver injury [34] (Fig. 5E). The influence of the above SNPs on the possible regulatory axis may be the molecular mechanism behind the positive genetic association results in this study. Specifically, HCC patients with rs1134492 CC + TC genotype had higher AST and ALT levels and worse prognosis compared to those with TT genotype, HCC patients with rs10589312 TCTTGC/TCTTGC + TCTTGC/T genotype had higher AST/ALT ratios than those with TT genotype, and patients with rs84557 TT genotype had higher AST/ALT ratios than those with CC + CT genotype.

In recent years, three lncRNAs located by the five positive association SNPs found in this study, namely *HOTAIR*, *PVT1* and *EGFR-AS1*, have been reported to be involved in the occurrence and development of a variety of cancers through the ceRNA mechanism. For example, it has been found that *HOTAIR* acts as an endogenous "sponge" for miR-148b to regulate the expression of DNMT1/MEG3/p53 pathway in hepatic stellate cells, which is related to the occurrence of HCC [35]. Another study showed that lncRNA *PVT1*, as a ceRNA, could compete with Atg3 to bind to microRNA-365 and promote autophagy in HCC cells [36]. Recently, lncRNA *EGFR-AS1* was found to be associated with migration, invasion and apoptosis of glioma cells by targeting miR-133b/RACK1 [37].

Several limitations of this study should be considered. First, we recommend that the findings of this association study should be expanded to other ethnic groups in different regions in the world, beyond the northeastern Chinese Han population. Second, the five potential regulatory axes of HCC related ceRNA affected by positive SNPs need to be verified by further in vitro and in vivo experiments.

## Conclusions

This is the first study to show that SNPs on lncRNA-mediated ceRNAs are associated with the HCC risk and prognosis. *HOTAIR* rs7958904, *PVT1* rs3931282, rs1134492 and rs10589312, and *EGFR-AS1* rs84557 might be involved in the occurrence or prognosis of HCC. The reason why these positive SNPs were associated with HCC are also explained from the perspective of the regulatory axis of ceRNA.

## Methods

### Data acquisition and identification of differentially expressed genes

In this study, liver hepatocellular carcinoma (LIHC) RNA-seq data (lncRNA and mRNA, level 3; including 374 HCC samples and 50 normal samples, Illumina HiSeq RNA-Seq platform) and miRNA-seq data (including 375 HCC samples and 50 normal samples, Illumina HiSeq miRNA-Seq platform) were downloaded from TCGA (https://tcga-data.nci.nih.gov/) in September 2019. The lncRNA and mRNA gene symbol was annotated using the Ensembl database (http://www.ensembl.org/). First, prior to differential expression analysis, all unexpressed RNAs were excluded by removing all lines in the gene expression matrix with a mean value of less than or equal to 1. Then the DElncRNAs, mRNAs DEmRNAs and miRNAs DEmiRNAs between the HCC group and the normal group were identified by edgeR package [38], and multiple test correction was performed using Benjamini and Hochberg false discovery rate (FDR) [39]. The differential expression threshold was FDR < 0.5 and |log2FC| (fold change) > 1.5. In this study, the pheatmap package was used to draw the hierarchical clustering heat map of DElncRNAs, DEmiRNAs and DEmRNAs data in R software (version 3.5.3).

### Study population

In this study, 800 HCC patients were recruited from the inpatient department in the Harbin Medical University Cancer Hospital between January 2007 and December 2016. At the same time, 801 healthy controls were collected from the Physical Examination Center of the First Affiliated Hospital of Harbin Medical University. The healthy control group was frequency-matched with the HCC case group according to gender and age. In addition, all subjects must be stable residents of the area. "Stable residents" means that all subjects are Han Chinese from northeast China who have lived in Harbin for at least three generations.

The diagnosis of HCC was based on histological, combined with at least one positive HCC image on computed tomography (CT) or magnetic resonance imaging (MRI), sometimes combined with serum AFP analysis (> 400 ng/ml). And none of the patients received any chemotherapy or radiation therapy before sampling. Moreover, patients with positive laboratory tests for HIV, or suspected autoimmune diseases with antinuclear antibody titer greater than 1:160 were excluded from this study. Each healthy control individual underwent the examination of antigen and antibody, at least one typical morphologic finding from CT or ultrasound. They were with no primary liver cancer, hepatitis, liver cirrhosis, or hepatic distomiasis.

### Sample collection information

Demographic data on participants were collected through in-person interviews, including age, gender, smoking and drinking status. Clinical characteristics of HCC patients were collected from patient medical records, including HBsAg status, anti-HCV, liver cirrhosis, Child–Pugh grade, tumor size, tumor number, vascular invasion, lymphatic metastasis, distant metastasis and TNM stage. Clinical test indexes such as AFP, AST, ALT and AST/ALT ratio in HCC patients were all obtained from the hospital patient information management system. Approximately 2–3 ml of venous blood samples were collected from each subject. Peripheral blood DNA was extracted by the QIAamp DNA Blood Kit (Valencia, CA, USA) and stored at -80 °C.

### Candidate SNP assessment and genotyping

Prior to genotyping, 22 candidate SNPs were evaluated to identify those that could not be successfully genotyped in the genotyping system and those with high linkage. For the former, the subsequent genotyping was discarded. For the latter, one SNP was selected as a representative from the highly linked SNPs. Finally, 15 SNPs were genotyped using a custom designed SNPscan™ kit based on the high-throughput SNP genotyping method using double ligation and multiplex fluorescent PCR. In a 200 μL PCR tube, LDR was performed in a final volume of 10 μL containing 3 μL of PCR products, 1 μL 10 × Taq DNA ligase buffer, 0.01 μL of each probe, and 5 U of Taq DNA ligase (NEB, USA). The reaction mixtures were heated for 2 min at 95 °C, followed by 30 thermal cycles at 94 °C for 30 s (denaturation) and 56 °C for 3 min (annealing and ligation). Prior to polyacrylamide gel electrophoresis, the 1 μL LDR products were mixed with 8 μL HiDi (highly deionized-formamide) and denatured for 3 min at 95 °C. The LDR products were separated by electrophoresis using the ABI 3730XL Gene sequencer. All PCR primers and gene probes used in PCR and LDR were designed using Primer 5.0 and Oligo 6.0 software. In terms of quality control, genotyping was performed twice in 5% of case and control random samples (a total of 80 samples) to verify the accuracy of genotyping, and the repeatability of genotyping results was 100%.

### Statistical analysis

The Pearson’s χ^2^ test was used to evaluate HWE for controls. Student's t test (for continuous variables) and χ^2^ test (for categorical variables) were conducted to compare the demographic characteristics between HCC cases and healthy controls. Among them, P-P plots and Levene’s test were used to assess the normal distribution and homogeneity of variance of continuous variables, respectively. The adjusted odds ratios (ORs) with their 95% confidence intervals (CIs) for the association between genotype frequencies and the risk of HCC plus clinicopathological characteristics (in HCC patients) were evaluated by multiple logistic regression models after controlling for other covariates. Meanwhile, the analysis of polymorphisms and biochemical indicators in HCC cases was performed by Mann–Whitney U test. Statistical analysis was conducted using SPSS version 25.0 software (SPSS, Chicago, IL, U.S.A.). All tests were two-sided and *P*-values less than 0.05 were considered statistically significant. Moreover, the method of FDR was used for multiple test correction.

### Acquisition of miRNA target genes and module analysis

In order to identify the effect of statistically significant positive SNPs in genetic association analysis on the potential ceRNA regulatory axis of HCC, target mRNAs of related miRNAs were extracted from miRWalk [40] and miRTarBase [41] databases. In order to improve the reliability of the results, only miRNA-mRNA relationship pairs supported by both databases were extracted. Then the online STRING database (https://string-db.org/) was used to construct the protein–protein interaction (PPI) networks for these targets. Interactions with a binding score > 0.4 were considered statistically significant.

The molecular interaction network was visualized using Cytoscape software (version 3.5.1), an open-source bioinformatics software platform. Cytoscape's Molecular Complex Detection (MCODE) plug-in (version 1.4.2) [42] was used to identify functional modules through cluster analysis to obtain the PPI network through topological network analysis. Thus, a protein complex with biological significance or functional module was obtained. The parameters are set as follows: Include Loops = false, Degree Cutoff = 2, Node Score Cutoff = 0.2, Haircut = true, Fluff = false, K Core = 2, Max. Depth from Seed = 100.

### KEGG and GO enrichment analyses of module genes

Kyoto Encyclopedia of Gene and Genome (KEGG) [43] and Gene Ontology (GO) [44] were mainly used to annotate and analyze biological processes (BP) of genes. In this study, ClueGO (version 2.5.7) [45] and CluePedia (version 1.5.7) [46] were selected to perform KEGG or GO-BP enrichment analysis on the most important modules in the module analysis. The parameters were set as follows: Show only Pathways with pV ≤ 0.01, GO Tree Interval Min Level = 4, GO Tree Interval Max Level = 7, Kappa Score = 0.6, Layout = yFiles Organic Layout.

## Supplementary Information

**Additional file 1:** **Supplementary Figure 1. **Genotyping feasibility assessment of candidate SNPs. Priority "Fail" was not genotyped, and one of the linkage SNPs (red) was selected for genotyping. **Supplementary Figure 2. **P-P plot shows that the age is normally distributed. The data points all fall on the obliqueline of 45°, showing strong normality.  **Supplementary Figure 3. **Association analysis of 14 SNPs with clinical test indexes in HCC patients. (A) Association analysis of 14 SNPs with AFP in HCC patients. (B) Association analysis of 14 SNPs with AST in HCC patients. (C) Association analysis of 14 SNPs with ALT in HCC patients. (D) Association analysis of 14 SNPs with AST/ALT ratios in HCC patients. Model 1 is the TCTTGC/TCTTGC+TCTTGC/T genetic model of rs10589312. Model 2 is the CC+C/CA genetic model of rs11438260. The values in red italics are statistically significant. The size of the indicator is represented by the median value (inter quartile range).

**Additional file 2:** **Supplementary Table 1. **Independent sample test of age.

**Additional file 3:** 

**Additional file 4:**

## Data Availability

The original contributions presented in the study are included in the article/Supplementary Materials, further inquiries can be directed to the corresponding author.

## Abbreviations

HBV: Hepatitis B virus
HBsAg: Hepatitis B surface antigen
HCV: Hepatitis C virus
HCC: Hepatocellular carcinoma
ALT: Alanine aminotransferase
AST: Aspirate aminotransferase
AFP: α-Fetoprotein
BP: Biological process
eRNA: Competing endogenous RNA
FDR: False discovery rate
GWAS: Genome-Wide Association Study
HWE: Hardy-Weinberg equilibrium
lncRNA: Long non-coding RNA
MAF: Minor allele frequency
miRNA: MicroRNA
OR: Odd ratio
PPI: Protein-protein interaction
SNP: Single nucleotide polymorphism
