# Supplementary material for: The effect of SNPs in lncRNA as ceRNA on the risk and prognosis of hepatocellular carcinoma
Source: BMC Genomics. 2022 Nov 24;23:769. doi: 10.1186/s12864-022-09010-9 (PMC9685961; doi:10.1186/s12864-022-09010-9)
Supplement: Supplementary file 1 — Additional file 1: Supplementary Figure 1. Genotyping feasibility assessment of candidate SNPs. Priority "Fail" was not genotyped, and one of the linkage SNPs (red) was selected for genotyping. Supplementary Figure 2. P-P plot shows that the age is normally distributed. The data points all fall on the obliqueline of 45°, showing strong normality. Supplementary Figure 3. Association analysis of 14 SNPs with clinical test indexes in HCC patients. (A) Association analysis of 14 SNPs with AFP in HCC patients. (B) Association analysis of 14 SNPs with AST in HCC patients. (C) Association analysis of 14 SNPs with ALT in HCC patients. (D) Association analysis of 14 SNPs with AST/ALT ratios in HCC patients. Model 1 is the TCTTGC/TCTTGC+TCTTGC/T genetic model of rs10589312. Model 2 is the CC+C/CA genetic model of rs11438260. The values in red italics are statistically significant. The size of the indicator is represented by the median value (inter quartile range). [file 12864_2022_9010_MOESM1_ESM.docx]

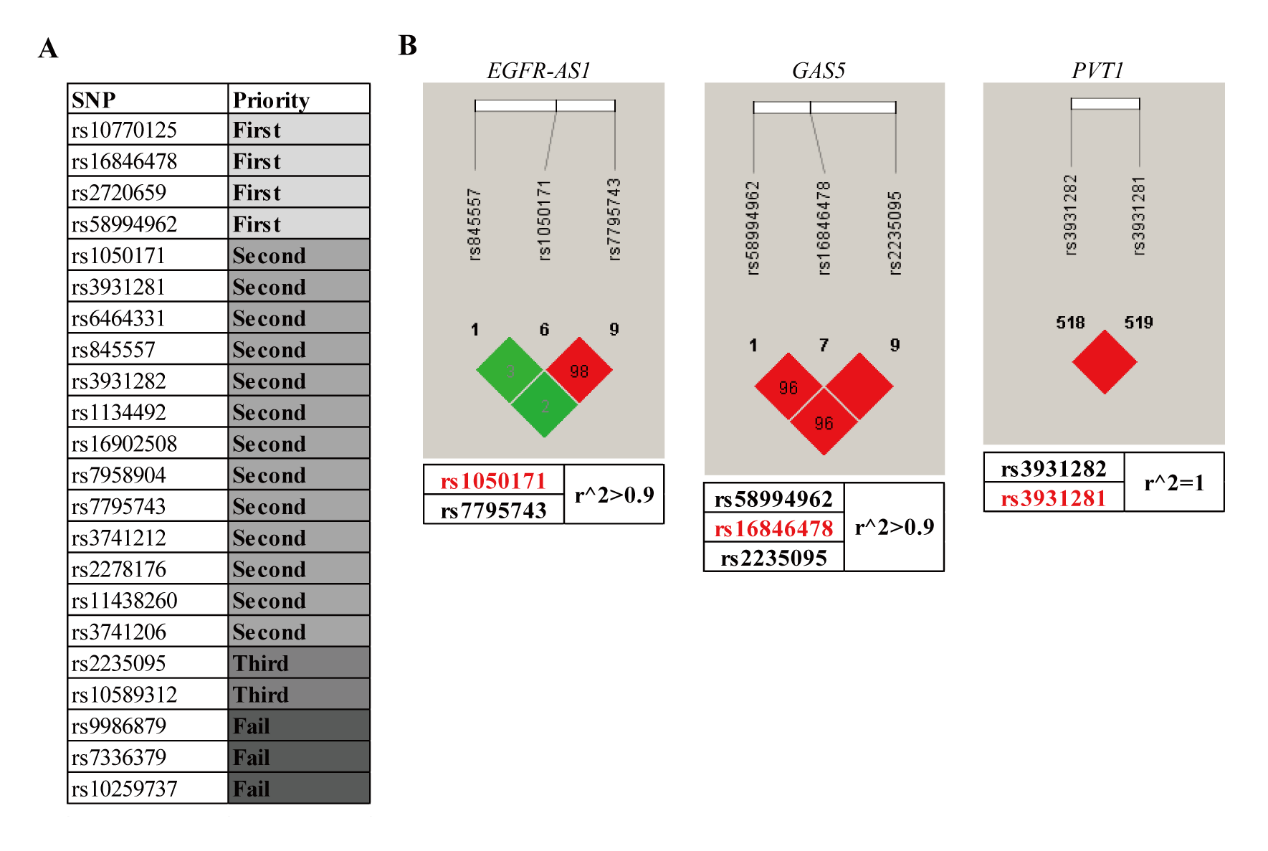
**Supplementary Figure 1. Genotyping feasibility assessment of candidate SNPs.** Priority "Fail" was not genotyped, and one of the linkage SNPs (red) was selected for genotyping.


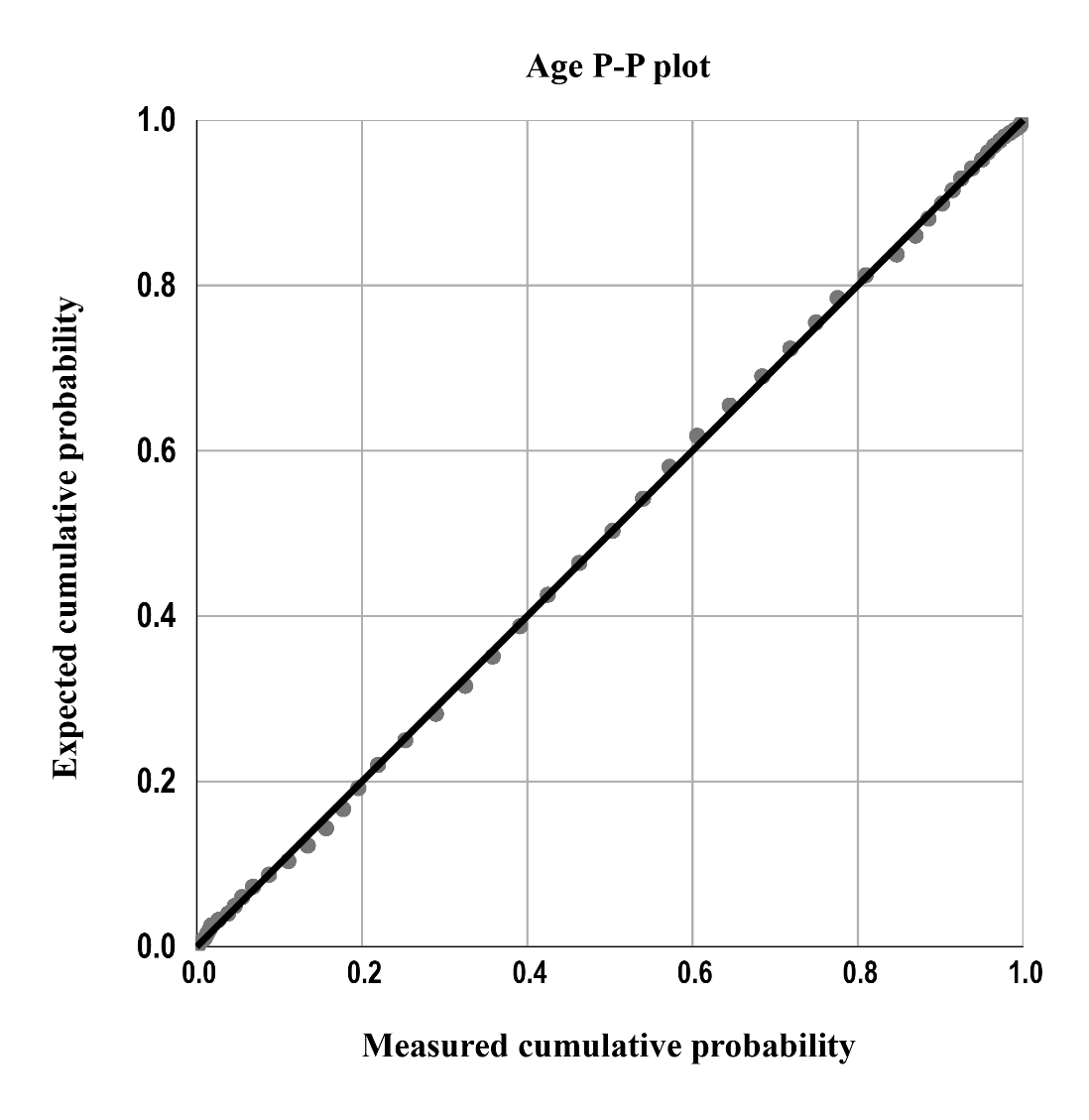
**Supplementary Figure 2. P-P plot shows that the age is normally distributed**. The data points all fall on the oblique line of 45°, showing strong normality.
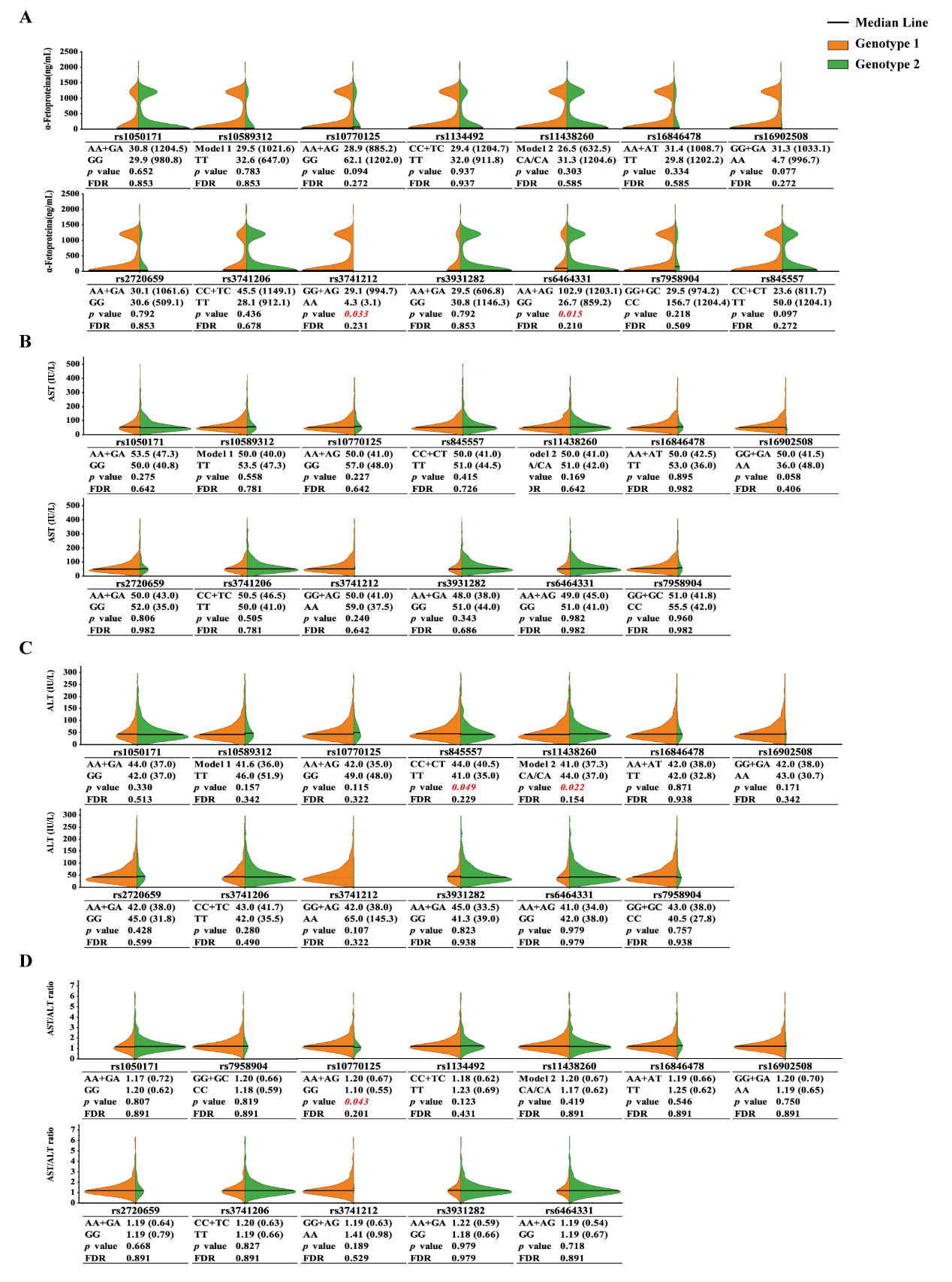
**Supplementary Figure 3. Association analysis of 14 SNPs with clinical test indexes in HCC patients.** (A) Association analysis of 14 SNPs with AFP in HCC patients. (B) Association analysis of 14 SNPs with AST in HCC patients. (C) Association analysis of 14 SNPs with ALT in HCC patients. (D) Association analysis of 14 SNPs with AST/ALT ratios in HCC patients. Model 1 is the TCTTGC/TCTTGC+TCTTGC/T genetic model of rs10589312. Model 2 is the CC+C/CA genetic model of rs11438260. The values in red italics are statistically significant. The size of the indicator is represented by the median value (inter quartile range).
