# Supplementary material for: The effect of SNPs in lncRNA as ceRNA on the risk and prognosis of hepatocellular carcinoma
Source: BMC Genomics. 2022 Nov 24;23:769. doi: 10.1186/s12864-022-09010-9 (PMC9685961; doi:10.1186/s12864-022-09010-9)
Supplement: Supplementary file 2 — Additional file 2: Supplementary Table 1. Independent sample test of age. [file 12864_2022_9010_MOESM2_ESM.docx]

**Supplementary Table 1. Independent sample test of age.**

|  | | Levene's Test for Equality of Variances | | t-test for Equality of Means | | | | | | |
| --- | --- | --- | --- | --- | --- | --- | --- | --- | --- | --- |
|  |  | F | Sig. | t | df | Sig. (2-tailed) | Mean Difference | Std. Error Difference | 95 % Confidence Interval of the Difference | |
|  |  |  |  |  |  |  |  |  | Lower | Upper |
| Age | Equal variances assumed | 0.000 | 0.988 | -0.188 | 1599.000 | 0.851 | -0.096 | 0.513 | -1.102 | 0.909 |
|  | Equal variances not assumed |  |  | -0.188 | 1599.000 | 0.851 | -0.096 | 0.513 | -1.102 | 0.909 |

Note: Levene's test for equality of variances, *P*=0.988, is considered as homogeneity of variance. See the first row of t-test results, that is, *P*=0.851.
